# Use of Tekscan K-Scan Sensors for Retropatellar Pressure Measurement Avoiding Errors during Implantation and the Effects of Shear Forces on the Measurement Precision

**DOI:** 10.1155/2013/829171

**Published:** 2013-12-03

**Authors:** A. Wilharm, Ch. Hurschler, T. Dermitas, M. Bohnsack

**Affiliations:** ^1^Department of Trauma, Hand and Reconstructive Surgery, University Hospital Jena, Erlanger Allee 101, 07747 Jena, Germany; ^2^Department of Orthopaedic Surgery, Medical School Hannover, 30625 Hannover, Germany

## Abstract

Pressure-sensitive K-Scan 4000 sensors (Tekscan, USA) provide new possibilities for the dynamic measurement of force and pressure in biomechanical investigations. We examined the sensors to determine in particular whether they are also suitable for reliable measurements of retropatellar forces and pressures. Insertion approaches were also investigated and a lateral parapatellar arthrotomy supplemented by parapatellar sutures proved to be the most reliable method. The ten human cadaver knees were tested in a knee-simulating machine at a torque of 30 and 40 Nm. Each test cycle involved a dynamic extension from 120° flexion. All recorded parameters showed a decrease of 1-2% per measurement cycle. Although we supplemented the sensors with a Teflon film, the decrease, which was likely caused by shear force, was significant. We evaluated 12 cycles and observed a linear decrease in parameters up to 17.2% (coefficient of regression 0.69–0.99). In our opinion, the linear decrease can be considered a systematic error and can therefore be quantified and accounted for in subsequent experiments. That will ensure reliable retropatellar usage of Tekscan sensors and distinguish the effects of knee joint surgeries from sensor wear-related effects.

## 1. Introduction

Following Fukubayashi and Kurosawa's first publication about Fuji pressure-sensitive films (Fuji Photo Col, Ltd., Tokyo, Japan) in 1980 [1], Fuji pressure-sensitive films were used for 15 years in biomechanical experiments to measure in vitro contact areas and pressures. Compared to modern technologies and systems, the uses of Fuji pressure-sensitive films were restricted due to their technical characteristics. Several tests showed measurement errors of Fuji films of between 10% [1, 2] and 15% [3]. In vitro measurements in knee joints could have errors between 14% and 28% [4].

Tekscan (Boston, MA, USA) pressure-sensitive films, introduced in the 1990s, have numerous advantages over Fuji sensors and have replaced them in biomechanical research [5–7]. Tekscan film sensors are offered in different sizes and shapes and consist of two electrically conductive sheets divided by an ink. An isolating grid creates a sensing location at each intersection. Forces applied to the sensors affect the thickness of the ink and its insulating effect. After calibration, one can measure the resistance at each intersection point and thus determine the applied force. Measurements can be taken continuously during an experiment. One can then determine the maximum force and maximum pressure, the force and pressure throughout the experiment, and the sensor's force area.

The purpose of our examination of Tekscan sensors was to prepare a series of experiments on changes in retropatellar pressure and force distribution after knee joint surgeries [8–10]. Prior to our studies, there were only a few publications which reported retropatellar pressure measurements using the Tekscan sensors for dynamic measurements [11–14]. Problems with using the sensors or the possible necessity of performing tests of the sensors durability have not been reported in the literature. For this reason, we performed a series of experiments to determine the optimal retropatellar implantation method as well as to detect possible sources of error when using Tekscan sensors. A further purpose was to record and quantify possible sensor wear in order to account for bias wear might cause in further studies which were planned [8].

## 2. Materials and Methods

**Figure 1:**
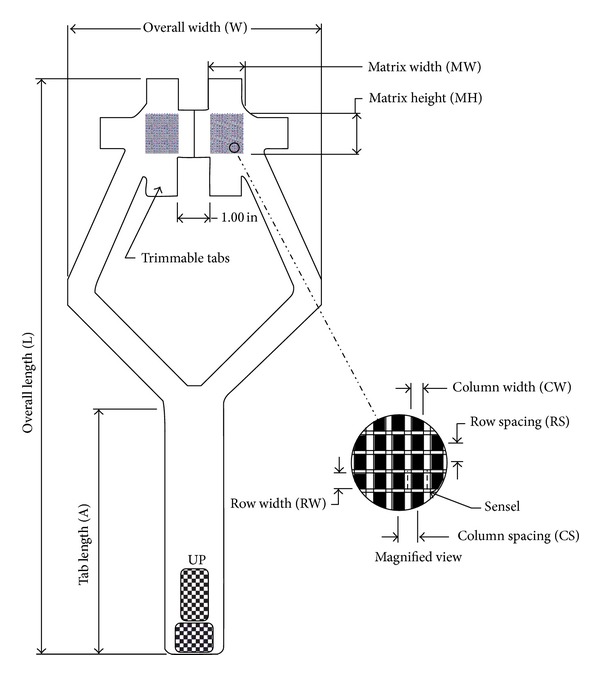
Drawing of a Tekscan 4000 sensor (copied by Tekscan, Inc., Boston, MA, USA).

The tested pressure measurement system (K-Scan 4000, Tekscan Inc., Boston, MA, USA) consists of the sensor, a transmission hardware (cable and sensor-interface or so-called handle), and a personal computer with the corresponding software (K-Scan Software, Version 4.23, Tekscan, Inc. Boston, MA, USA). The employed sensors are 0.1 mm thin and contain 572 individual sensor-elements (62 per cm^2^) which are oriented along 26 rows and 22 columns (Figure 1, Table 1). Each sensor covers a measurement area of 22 × 33 mm or 924 mm², with a resulting spatial resolution per sensel of 1.02 × 1.27 mm (Table 2). Prior to use, sensors were preconditioned and calibrated according to the manufacturer's recommendations using a material testing machine (Mini Bionix 858, MTS, Eden Prairie, MN, USA). During this procedure the sensors were placed between two aluminium cubes such that each sensor area was almost completely covered. A silicon-rubber sheet of approximately 2 mm thickness was used to distribute the loading and prevent the saturation of individual sensls or senesel areas. In a first step the sensors were preconditioned by applying 100 cycles of 5000 N loading and subsequently dynamically calibrated at 0 N, 2500 N and 5000 N.

Biomechanical evaluation of the sensors was performed on 10 human cadaver knee specimens (6 male, 4 female, average age of 44 years). After removing skin and subcutaneous tissue, the tendon of the quadriceps femoris muscle was dissected free of muscle tissue along its entire length and fixed in a specially constructed tendon clamp. The femur and tibia were rigidly attached to their respective attachment points in a dynamic loading simulator using bone cement (Palacos, Heraeus Medical GmbH, Hanau, Germany) (Figure 3).

A dynamic physiologic-loading simulator according to Hofmann et al. [15] was used to simulated extension motions of the of the knee joint from approximately 120° flexion to full extension, similar to the motion occurring when a subject moves from a deep squatting to a standing posture. Based on the work of Hassenpflug and Shinno, load was applied through the quadriceps tendon 2° lateral to the femur roll axis [16, 17]. The knee-extension moment was 30 and 40 Nm.

In stage I of the experiments, using anatomic specimens, we determined the optimal implantation method for the sensors as well as the best access path for the sensors. To do so, we examined various specific adhesives and degreasing solutions, as well as several suturing techniques for fixing the sensors. First, we tested the stability of the sensors by hand and performed loading-cycles in the simulator to identify any loosening or slipping of the sensor film. In addition, we tested several access paths for the sensors, and we examined whether contact with the data bus had a noticeable effect on results. During the tests we evaluated the needed access size as well as its effects on the sensor films and the alignment of the patella.

In stage II of the experiments, we implanted each of the sensors via the method determined in stage I. Without any alteration of the test setup or the knee joint, we conducted two rounds of 3 cycles with a knee-extension moment of 30 and 40 Nm. The parameters measured were recorded at rate of 10 Hz (force, contact area, pressure, maximal force, and maximal pressure). All recorded parameters were evaluated statistically using the Wilcoxon signed-rank test (SPSS version 11.0.1, SPSS inc., Chicago, USA).

## 3. Results

**Figure 2:**
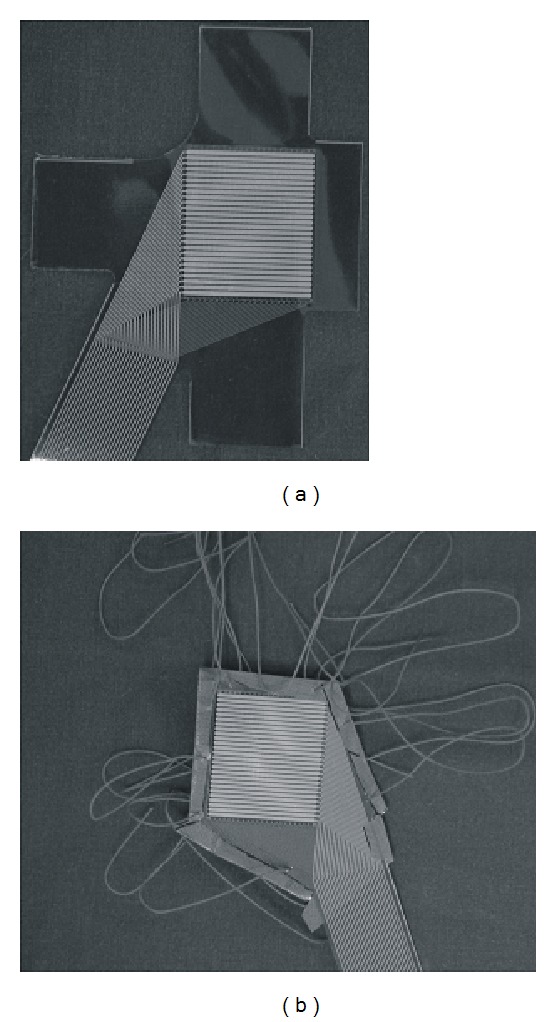
Preparation of the sensors for implantation ((a) original sensor, (b) after augmentation with a Teflon film and sutures).

**Figure 3:**
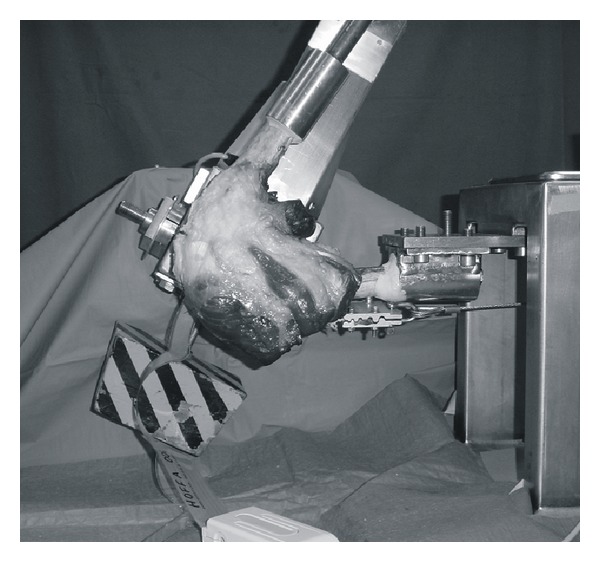
Knee specimen mounted in the knee-simulating machine.

Our initial tests revealed that it was not possible to affix the sensor film properly on the rear patellar area using adhesives only. None of the tested adhesives adhered sufficiently to the cartilage's extremely smooth surface. Cauterization (if done with heat) or etching (if done with chemicals) or roughening of the cartilage's surface was not considered as its shape and characteristics would have been changed. Parapatellar sutures (Vicryl size 1.0, Ethicon, Norderstedt, Germany) and a supplementation of the sensors with a 0.1 mm Teflon film proved to be the most stable method for fixing the sensors in location and preventing their movement during testing (Figure 2). The method finally used involved stabilizing the sensor by transecting the tissue adjacent to the patella with a surgical needle and attaching each of the 8 respective suture ends to their adjacent suture neighbour. Sutures that were not attached to one another and simply tied to the surrounding soft tissue proved to be insufficiently stable to guarantee fixation of the sensor.

The Teflon film reinforcement was found to be necessary to increase the durability of the sensors in this application to a degree which permitted reliable measurements to be made (Figure 2). With this modification, it was possible to perform 4 premeasurement cycles and 12 measurement cycles without any failures of sensor rows [9]. Without the Teflon film complete sensor rows failed after only a few cyclic movements.

A lateral, 4 cm long parapatellar miniarthrotomy proved to be the most convenient access path for implantation. It enabled us to perform the sutures properly as well as to pull the sensor into place reliably. With this approach it was possible to rout the leads connecting the sensor to the interface handle through the same access path without any cases of breakage. Medial parapatellar access led to the weakening of the medial retinaculum and patellaluxation. A lateral inferior access was not employed because of interference of the infrapatelar fat pad, which hindered observation and prevented implantation of the sensor.

Evaluating the data from 16 measurement cycles revealed that they must be divided into premeasurement cycles and measurement cycles which could evaluated statistically. While recorded parameters of the first 2 premeasurement cycles showed significant variations, the parameters associated with the following 12 measurement cycles parameters showed nearly identical curves of each cycle. Nonetheless, we did observe a continuous decrease of recorded parameters of 1–2% per cycle (Table 2).

At a torque of 30 Nm the force measured declined by an average of 3.3% after 3 measurements cycles and by 12.3% after 9 cycles. The load area declined by 1.5% after 3 cycles and by 7.6% after 9 cycles. At a torque of 40 Nm, force declined by 2.6% after 3 cycles and by 11.1% after 9 cycles; the load area declined by 1.8% after 3 cycles and by 6.2% after 9 cycles. These changes represent changes in the pressure-measurement system since neither the test setup and hence the actually occurring loading situation nor the knee joints themselves were altered during these repeated measurements.

An analysis of the changes in the reported parameters revealed that the changes were occurring in a nearly linear manner in dependence on the number of loading cycles. The coefficient of regression (*r* ^2^) was observed to be between 0.96 and 0.99 (Figure 5).

## 4. Discussion

The piezoresistive pressure measurement technology has presented new possibilities for the dynamic measurement of force and pressure in biomechanical investigations. The sensors were developed for measuring axial loads on generally flat surfaces. Correspondingly, Tekscan Inc. has performed respective tests for accurate measurements. The need for correct calibration of the sensors was noted in the literature [5, 18–20]. The number of calibration points influences measurement accuracy. Increasing the number of points from 3 to 11 improved measurement accuracy significantly and reduced the deviation in measurements from 14% to 4% [18]. When applying axial force, it was possible to reduce the deviation further of 0.6%, with a specific calibration protocol. We recommend executing the tests immediately after calibration; after sensors were stored for 1 week the measured force decreased by 3.4% [19]. In addition, we recommend maintaining the same temperature between calibration and testing [21]. Finally, the sensors should be sterilised before calibrating as sterilisation causes a decalibration. However, sterilisation does not affect measurement accuracy [19]. Recently, Jansson et al. observed a linear reduction of load output over time in the presence of liquid saturation over several hours [22]. This could not be the explanation for the sensor output changes we observed because in our application the sensors were not in contact with liquids for more than 10 to 15 minutes. Changes of the viscoelastic tissue response are unlikely to be the cause of the linear decrease in each parameter we observed, because of the short time we needed for the measurements and relatively small number (16) of measurement cycles performed per knee.

A further purpose of our study was to determine the optimal access path for implantation of the sensors and a reliable method for attaching the sensor film to the convex geometry of the retropatellar joint surface. We also sought to determine whether the presence of shear force in a typical dynamic-loading simulation causes errors in measurements or failures of sensor films.

**Figure 4:**
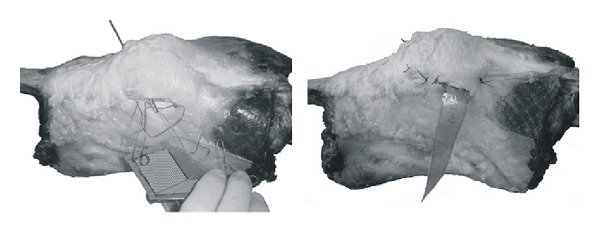
Implantation of the sensor through a proximal lateral access.

A parapatellar lateral miniarthrotomy proved to be the most convenient access path where we placed the sutures and stabilized the sensor properly. During testing we did not observe any opening of the arthrotomy through which the sensor leads were routed (Figure 4). By contrast, a medial arthrotomy led to a decentralisation of the patella with numerous patellar luxations. Hence, it cannot be considered for implantation of the sensors nor for investigations during testing because it changes the biomechanics of the patello-femoral joint and thus probably also the kinetics and kinematics of the entire knee joint.

Measurement accuracy relies on the secure attachment of the sensor to the retropatellar joint surface. All measured parameters are useless when the sensor position shifts during the test. Anderson et al. reported that the results of complete tests could not be evaluated because of shifts in sensor position [23]. As determined in our experiments, parapatellar sutures provide a reliable implantation method for sensors. A circular mounting of sutures around the patella made shifts in sensor position impossible. A more stable, purely transossary sensor implementation (i.e., integrating a sensor technology into the patellar bony structure) would require a more invasive approach including a mobilisation of the patella and destruction of the retinacula which would most probably lead to alterations of the gliding mechanism.

In our opinion, it is undesirable to switch sensors during a test because it is difficult to ensure repeatable measurements under such conditions. Thus, when investigating the effects of a surgery on patellofemoral biomechanics, the sensor should remain in situ and should not be shifted from its position.

The durability and repeatability of Tekscan sensors are considerably restricted in the presence of shear force. While a pure axial load does not affect measurement results, patellofemoral tests lead to the failure of complete sensor rows after 17–20 cycles. In our assessment, this is due to a rupture of the various sensor films from the effects of shear force. Thus, we were forced to restrict our tests to 16 measurement cycles, and the first 4 premeasurement cycles were not included in our evaluation. A supplementation of the sensors with a 0.1 mm Teflon film improved their durability. Tekscan Inc. supplies that film on request.

**Figure 5:**
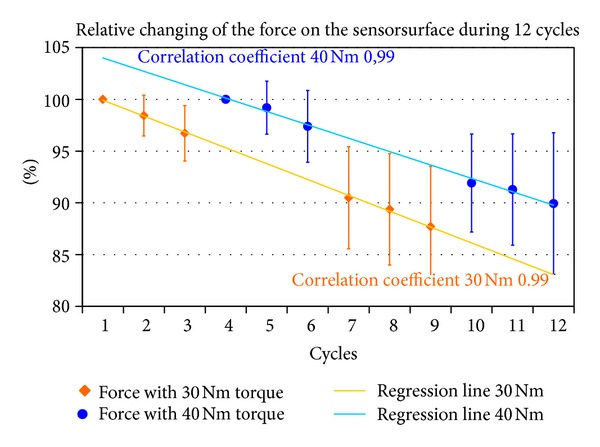
Relative decrease of the patellofemoral force over 12 measurement cycles with linear regression line for the measurements at 30 and 40 Nm. The first measurement cycle at 20 and 40 Nm is shown as 100%. Measurement cycles 1–3 and 7-8, 30 Nm torque. Measurement cycles 4–6 and 10–12, 40 Nm torque.

**Figure 6:**
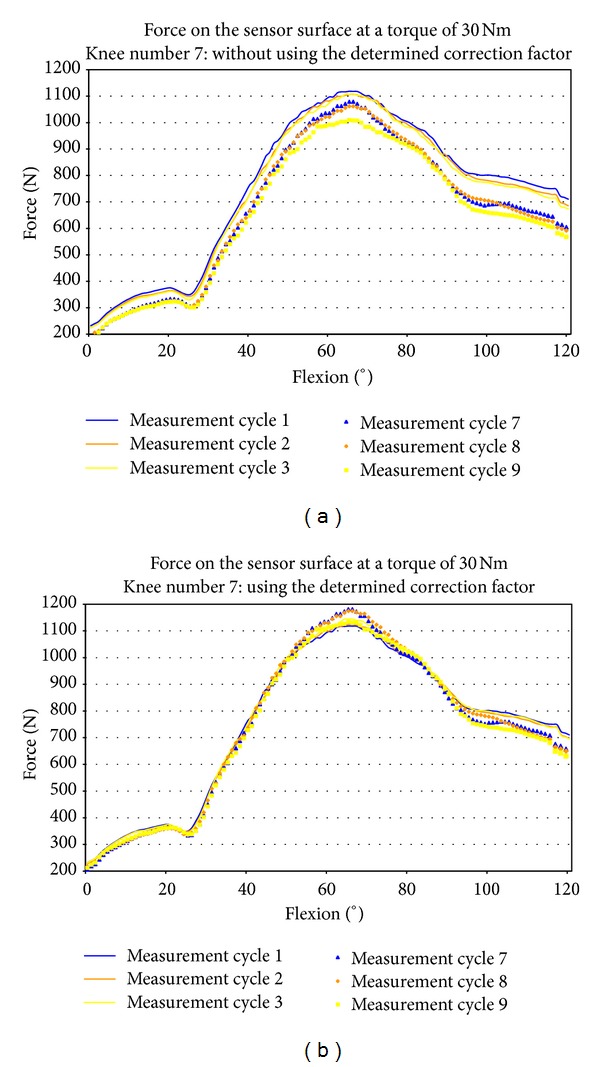
Exemplary application of the calculated average correction factor of 10 knees on the results of knee number 7. Above distinguishable change in force during the course of several measurement cycles without changing the experimental setup. Below using the determined correction factor the systematic error is barely recognizable.

Pavlovic et al. noted an error in measurement of 2.3% after over 30 test cycles with the tibiofemoral joint [24]. By contrast, our measurements in the patellofemoral joint over 12 cycles show a linear decrease in each parameter of 1-2% per cycle (Figure 5, Table 2). Accordingly, at a torque of 30 Nm, a total decrease in contact area of 12.7% can be derived, as can a decrease in force of 17.2%. In our opinion, the linear decrease of parameters can be considered a systematic error. Consequently, that decrease can be quantified and accounted for in subsequent experiments using the same test protocol (Figure 6). We have taken the approach that a correction based on calibration-loss curves from preliminary experiments is used to correct pressure measurements performed during repeated loading scenarios. We feel that a quantification of this systematic error is essential in order to ensure reliable retropatellar usage of Tekscan sensors, and in particular to distinguish between the effects of knee joint surgeries and sensor wear-related effects leading to calibration degradation. When used in this context, this measurement method is appropriate for the scientific evaluation of various surgical procedures, for example, lateral retinacular release, tibial tuberositas transfer, and so forth [13, 14, 25, 26] and the development of knee joint endoprosthesis [27–29].

## 5. Conclusions

It is possible to measure the retropatellar pressure using Tekscan K-Scan 4000 sensors. To obtain reliable measurement results, a rigid retropatellar fixation of the sensors as well as pretests to quantify the systematic error coursed by wearing of the sensors is essential.

## Figures and Tables

**Table 1:** The K-Scan 4000 sensors.

Overall length *L*	Overall width *W*	Tab length *A*	Matrix width MW	Matrix height MH	Columns			Rows			Total no. of sensels	Resolution
					CW	Pitch CS	Qty.	RW	Pitch RS	Qty.		Sensel density
(in.)	(in.)	(in.)	(in.)	(in.)	(in.)	(in.)		(in.)	(in.)			(sensel per in.²)
18.19	8.00	7.80	1.10	1.30	0.030	0.050	22	0.040	0.050	26	572	400.0
(mm)	(mm)	(mm)	(mm)	(mm)	(mm)	(mm)		(mm)	(mm)			(sensel per cm²)
462.0	203.2	198.1	27.9	33.0	0.8	1.3	22	1.0	1.3	26	572	62.0

**Table 2:** Percentage decrease of the measured retropatellar parameters in 12 consecutive measurement cycles without changing the experimental setup.

	Torque 30 Nm					
	Cyclus 1	Cyclus 2	Cyclus 3	Cyclus 7	Cyclus 8	Cyclus 9
Force	0%	−1.6% ± 1.3	−3.3% ± 1.4	−9.5% ± 4.7	−10.6% ± 4.1	−12.3% ± 3.9
Area	0%	−0.7% ± 1.0	−1.5% ± 0.9	−5.9% ± 4.2	−6.2% ± 3.4	−7.6% ± 2.9
Pressure	0%	−1.0% ± 0.7	−1.8% ± 1.0	−3.8% ± 2.1	−4.6% ± 2.0	−5.0% ± 2.1
Max. force	0%	−1.2% ± 1.0	−2.2% ± 1.3	−3.8% ± 2.2	−4.5% ± 2.4	−5.1% ± 2.4
Max. pressure	0%	−1.1% ± 0.9	−2.1% ± 1.2	−3.8% ± 2.2	−4.4% ± 2.4	−5.0% ± 2.4

	Torque 40 Nm					
	Cyclus 4	Cyclus 5	Cyclus 6	Cyclus 10	Cyclus 11	Cyclus 12

Force	0%	−0.8% ± 1.0	−2.6% ± 1.9	−8.1% ± 3.1	−8.7% ± 1.8	−10.1% ± 3.1
Area	0%	−0.7% ± 0.9	−1.8% ± 2.1	−4.2% ± 2.4	−5.2% ± 2.3	−6.2% ± 1.7
Pressure	0%	−0.2% ± 0.8	−1.0% ± 0.8	−3.7% ± 2.2	−3.8% ± 1.8	−4.5% ± 2.6
Max. force	0%	−0.3% ± 1.7	−1.2% ± 2.1	−2.8% ± 1.4	−3.5% ± 1.9	−4.2% ± 2.9
Max. pressure	0%	−0.3% ± 1.6	−1.2% ± 2.1	−2.8% ± 1.4	−3.4% ± 1.9	−4.2% ± 2.9
